# Intercropping with *Achyranthes bidentata* alleviates *Rehmannia glutinosa* consecutive monoculture problem by reestablishing rhizosphere microenvironment

**DOI:** 10.3389/fpls.2022.1041561

**Published:** 2022-11-22

**Authors:** Yazhou Liu, Ye Liu, Chunli Zeng, Juanying Wang, Witness Joseph Nyimbo, Yanyang Jiao, Linkun Wu, Ting Chen, Changxun Fang, Wenxiong Lin

**Affiliations:** ^1^ Fujian Provincial Key Laboratory of Agroecological Processing and Safety Monitoring, College of Life Sciences, Fujian Agriculture and Forestry University, Fuzhou, Fujian, China; ^2^ Key Laboratory of Crop Ecology and Molecular Physiology, Fujian Agriculture and Forestry University, Fuzhou, China; ^3^ Key Laboratory of Plant Resources Conservation and Germplasm Innovation in Mountainous Region (Ministry of Education), Institute of Agro-bioengineering, College of Life Science, Guizhou University, Guiyang, Guizhou Province, China; ^4^ Guizhou Key Lab of Agro-bioengineering, Institute of Agro-bioengineering, College of Life Science, Guizhou University, Guiyang, Guizhou Province, China

**Keywords:** consecutive monoculture problem, intercropping, root exudate, rhizosphere microbe, allelopathy

## Abstract

**Background:**

The consecutive monoculture of *Rehmannia glutinosa* leads to a serious decrease in its production and quality. Previous studies have demonstrated that intercropping altered species diversity and rhizosphere microbial diversity. However, it remained unknown whether the impaired growth of monocultured plants could be restored by enhanced belowground interspecific interactions.

**Method:**

In the present research, a continuous cropping facilitator *Achyranthes* bidentata was intercropped with *R. glutinosa* under pot conditions, and three different types of root barrier treatments were set, including that complete belowground interaction (N), partial belowground interaction (S), and no belowground interspecies interaction (M), with the aims to investigate belowground interaction and the underlying mechanism of alleviated replanting disease of *R. glutinosa* by intercropping with *A. bidentata*.

**Results:**

The results showed that the land equivalent ratio (LER) of the two years was 1.17, and the system productivity index (SPI) increased by 16.92 % under S treatment, whereas no significant difference was found in N and M regimes. In the rhizosphere soil, intercropping systems had significantly increased the contents of sugars and malic acid in the soil of *R. glutinosa*, together with the content of organic matter and the invertase and urease activities. Meanwhile, intercropping increased the community diversity of fungi and bacteria, and the relative abundance of potential beneficial bacteria, such as *Bacillus*, *Nitrospira*, and *Sphingomonas*, despite the pathogenic *Fusarium oxysporum* was still the dominant genus in the rhizospheric soil of *R. glutinosa* under various treatments. The results of antagonism experiments and exogenous addition of specific bacteria showed that *Bacillus spp.* isolated from rhizosphere soil had a significant antagonistic effect on the pathogen of *R. glutinosa*.

**Conlusion:**

Taken together, our study indicated that the *R. glutinosa//A. bidentata* intercropping systems alleviate the consecutive monoculture problem of *R. glutinosa* by recruiting beneficial bacteria. The studies we have conducted have a positive effect on sustainable agricultural development.

## Introduction

Traditional Chinese Medicine (TCM) is one of the oldest healing systems and it is an important aspect of Chinese healthcare (Tang et al., 2008). Herbs are the substances mostly used in TCM, and many of those drugs have shown dramatic effects in the treatment of diseases, such as *Artemisia annua* L. (Xu et al., 2019). Large-scale monocultures are becoming increasingly popular to meet market demands. However, continuous monocropping has also led to many problems, including an increased rate of disease incidence and a loss of crop yield and quality, which is also known as replanting disease or soil sickness. These problems are particularly acute in the cultivation of medicinal herbs, such as *Rehmannia glutinosa*, *Pseudostellaria heterophylla*, and *Panax ginseng* (Yin et al., 2009; Dong et al., 2018; Li et al., 2020; Chen et al., 2021; Liu et al., 2021). *R. glutinosa* plants belong to the family of Scrophulariaceae, a famous Chinese herbal medicine, with various pharmaceutically active compounds, contains iridoids, amino acids, and inorganic ions, et. (Zhang et al., 2008). It is produced mainly in Jiaozuo city, Henan Province, central China, which is recognized as the geo-authentic source in Chinese medicine. However, consecutive monoculture planting of *R. glutinosa* in the identical land led to a significant increase in root rot disease and a serious decrease in tuber production (Wu et al., 2015) (**Supplementary Figure 1**). Regulation of soil negative feedback for consecutive monoculture problems needs almost 15 years to be eliminated (Yang et al., 2011). In traditional Chinese medicine, geo-authentic medicinal herbs are considered to be one of the highest qualities in all samples from different regions. The replanting disease has significantly reduced the available arable land in the geo-authentic production zone, resulting in the migration of herb cultivation to non-geo-authentic production areas, which affects the quality of the herbs, which is detrimental to the sustainable development of Chinese herbs. Therefore, it is urgent to implement sustainable and ecological cultivation patterns to ensure the quality of medicinal materials and food security.

In recent years, numerous studies have suggested that belowground microbes play an essential role in plant growth, development, and health (Trivedi et al., 2020; Xiong et al., 2020). Rhizosphere microbes act as the second genome of plants enabling greater environmental adaptability, resource acquisition, defense reactions, and the communication between plants. However, previous studies suggested that replanting disease of Chinese herbs was attributable to changes in soil microbial community structure mediated by root exudates in continuous cropping system (Wu et al., 2017; Li et al., 2022; Xu et al., 2022). In addition, consecutive monoculture of *R. glutinosa* markedly enhanced the relative abundances of two major pathogenic fungi, *Fusarium oxysporum* and *Aspergillus flavus*, but decreased the relative abundances of potentially beneficial microorganisms genera *Pseudomonas*, *Burkholderia*, *Azotobacter*, among others (Wu et al., 2018b). Comparable cases have been found in a number of other medicinal plants, for instance, consecutively monocultured *Radix pseudostellariae* is known to be in charge of increasing the species size of pathogenic *Fusarium oxysporum* and *Talaromyces* helices and to reduce the population size of potentially beneficial bacteria *Pseudomonas* spp. and *Burkholderia* spp (Wu et al., 2017; Wu et al., 2019). However, a recent study found that consecutive monoculture of *Achyranthes bidentata* can be enriched with beneficial microbes, such as *Bacillus*, *Fictibacillus*, *Bradyrhizobium*, *etc.* through root exudates (Wang et al., 2019; Wang et al., 2021b). *A. bidentata* (family Amaranthaceae) is a commonly used Chinese herbal planted in the same geo-authentic production zone as *R. glutinosa.* However, *R. glutinosa* accumulates pathogens in its surrounding soil and forms negative soil feedback, but *A. bidentata* can increase the potentially beneficial microbes that produce soil memory or ‘soil-borne legacy’. This unbreakable relationship among plants and soil microbiome is important for plant health and productivity (Berendsen et al., 2012; Lapsansky et al., 2016; Ling et al., 2022). Therefore, it is suggested that the rhizosphere management of improving the rhizosphere microenvironment may be an effective strategy for the control of replanting disease.

Accordingly, it has been hypothesized that plants influence their abundances by changing the structure of their soil communities; negative soil feedback can regulate plant biodiversity, which is an important regulator of plant community structure (Klironomos, 2002; Berendsen et al., 2018). Therefore, a promising strategy has been adopted to alleviate replanting disease using biologically diversified farming to provide multiple ecosystem services. Meanwhile, in the process of soil memory formation, root exudates play an important role (Berendsen et al., 2018; Sasse et al., 2018; Yuan et al., 2018); the plants can release allelochemicals into the microenvironment of rhizosphere to suppress harmful soil-borne pathogens (Meiners et al., 2012). However, since the chemicals do not spread widely in soil, they can only have an impact in a limited area. Therefore, intercropping patterns between different plants are suitable for this type of direct pathogen suppression (Homulle et al., 2021). For instances, watermelon (*Citrullus lanatus* Trunb) rotated with rice can reduce *Fusarium* wilt disease of watermelon (Ren et al., 2008). Peanut intercropping with *Atractylodes lancea* effectively suppressed pathogenic *Fusarium* populations by using volatiles from the *A. lancea* rhizome, thereby increased the production of peanuts (Li et al., 2018). In the field experiment, researchers intercropped two different varieties of *R. pseudostellariae*, by which the replant disease could be alleviated (Wu et al., 2020). Therefore, root exudates from the interspecific plants in intercropping systems may alleviate replant diseases in agriculture by altering the composition of microbial community structure and changing the soil’s feedback regulation among the whole ecosystem through this domino effect. In the context of agroecosystems, if a particular function is desired, it can be beneficial to focus on a few efficient species supporting this function (Cappelli et al., 2022). In *in vitro* interactions, Wang et al. (2021a) found that the root exudate of *A. bidentata* showed a positive interaction with dominant beneficial species and had a detrimental impact on the existence of the pathogenic fungi *F.solani* and *F. oxysporum*. Therefore, is it a feasible strategy to alleviate the replanting disease of *R. glutinosa* by intercropping with *A. bidentata*?

In our previous study, we revealed that intercropping *R. glutinosa* with *A. bidentata* could reduce the effect of continuous cropping on the yield of *R. glutinosa*, which is related to the altered microenvironment in the underground after intercropping (data not available here). In addition, in intercropping systems, competitive underground activities usually come in various paths and involve in complex processes (Schenk, 2006). Therefore, to clarify the underlying mechanisms of belowground interactions and ecological relationships among the species in soil ecosystem, we adopted the root partitioning technique as described by previous studies (Wang et al., 2018) to uncover the scientific mysteries existing in the interspecific cropping system. In this study, we developed three root barrier treatment types to study subsurface interactions: no root barrier, a nylon mesh barrier, and a plastic film barrier. Using the *R. glutinosa* variety ‘Jing9’ and *A. bidentata* variety ‘HeTaoWen’, we first tested whether the intercropping of the two medicinal plant species was able to alleviate the replanting disease of *R. glutinosa* in the cropping system. Then, we analyzed the soil physicochemical and biological properties in this process. Finally, we analyzed the changes of interspecific-root microorganisms and root exudates under different barrier treatments to explore the effects of intercropping between *R. glutinosa* and *A. bidentata* in interspecific-root microenvironment, which aids the understanding the underlying mechanisms through the intercropping to alleviate the replanting disease of *R. glutinosa* by the other intercropping crop, *A. bidentata* medicinal plants in cropping system.

## Materials and methods

### Experimental site and soil sampling

In this study, the soil used for pot trial, was taken from a field in Xitao town, Wuzhi city, Henan Province, China (35.6 N, 113.22 E) in April 2020. The field had been used to plant *R. glutinosa* in past year. The site is recognized as the geo-authentic production zone of *R. glutinosa* and *A. bidentata*. In addition, the control soil was taken from the fields never planted with *R. glutinosa*. The upper soil samples within 20 cm layers were randomly collected and sifted to allow for removal of plant fragments and rocks, and then stored at ambient temperature. The soil pH was 8.2, which was sampled from and detected in the field of *R. glutinosa* monocultured for one year, and the contents of available nitrogen, potassium, and phosphorus were 128.33, 287.67, and 275.27 mg·kg^−1^, respectively. For the newly planted soil, the total nitrogen, potassium, and phosphorus were 0.54, 0.67, and 0.57 g·kg^−1^. The pH was 8.1, and the contents of available nitrogen, potassium, and phosphorus were 117.98, 282.89, and 270.86 mg·kg^−1^, and the total nitrogen, potassium, and phosphorus were 0.48, 0.56, and 0.62 g·kg^−1^, respectively.

The pot experiments were conducted at the agroecological experimental station of Fujian Agriculture and Forest University (26.5 N, 119.13 E), Fuzhou, Fujian Province, China. The mean annual precipitation in this region is 1415 mm and the mean annual temperature is 20.9°C. *R. glutinosa* cultivar ‘Jing9’ and *A. bidentata* cultivar ‘HeTaoWen’ were used as the experimental materials for this study. In this experiment, *R. glutinosa* (R) and *A. bidentata* (A) were grown in pots of a 1-year monoculture *R. glutinosa* soil, and three root partitioning patterns for the intercropping of *R. glutinosa* with *A. bidentata* plants were designed with three replications. Without root partition (i.e., belowground interactions existed, N), a nylon mesh barrier root partitioned (partial belowground interactions existed, S), and a plastic film root partition (no belowground interactions existed, M). Furthermore, *R. glutinosa* were grown alone in a plot with newly planted (FR) soil as control with three replications.

**Figure 1:**
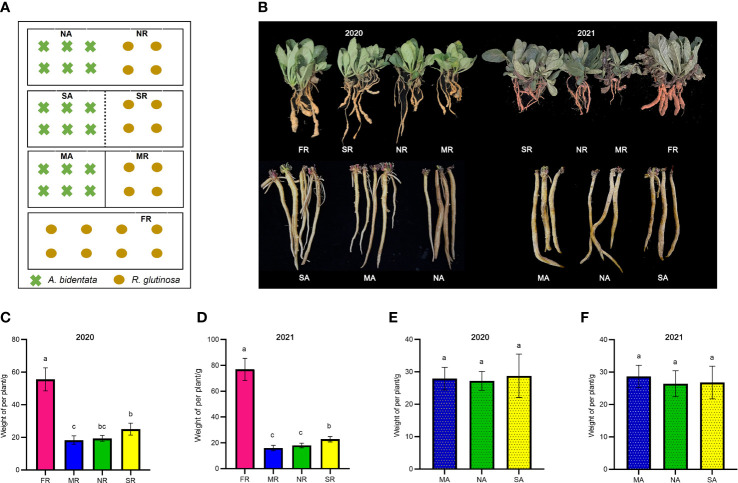
Phenotypic distribution of yield traits. The schematic diagram of experimental design **(A)**. The picture of plant growth in 2020 and 2021 **(B)**. The Fresh weight of *R. glutinosa* root in 2020 and 2021 **(C, D)**. The Fresh weight of **(A)** *bidentata* root in 2020 and 2021 **(E, F)**. FR, newly planted *R. glutinosa*; MR, plastic film barrier treatment *R. glutinosa*; MA, plastic film barrier treatment *A.bidentata*; SR, nylon mesh barrier treatment *R. glutinosa*; SA, nylon mesh barrier treatment *A.bidentata*; NR, no root barrier *R. glutinosa*; NA, no root barrier *A.bidentata*; the same below. Different letters in columns show significant differences determined by LSD’s test (p<0.05, n=3).

Based on the design, in the first year, *R. glutinosa* was planted on April 20 in 2020, and *A. bidentata* was planted on July 20 in 2020, and all were harvested on November 30, 2020; for the second year, *R. glutinosa* was planted on April 15 in 2021 and *A. bidentata* was planted on July 15 in 2021, and all harvested on December 2 in 2021. Each treatment had three experimental repetitions (0.78 m long × 0.39 m wide × 0.37 m high) (**Figure 1A**, **Supplementary Figure 2**). Each plot was subjected to the same fertilization and water management over the duration of the experiment. *R. glutinosa* was regarded as the major crop and *A. bidentata* as the intercrop component. The grain yield of *A. bidentata* from each plot was transformed into the equivalent yield of intercropping system as the Land Equivalent Ratio (LER) and System Productivity Index (SPI) (Agegnehu et al., 2006).

The samples were taken from each potted plot randomly, and the roots were dug out with a shovel to remove bulky soil, and then the soil with a thickness of about 2 mm on the surface of the roots was collected as rhizosphere soils. The three independently sampled soils were mixed into one sample and replicated three times. All soil samples were then sifted with a 2 mm sieve, and then some were kept at -80°C for soil total DNA extraction and metabolic extraction, and the others were stored at -20°C for determination of soil enzyme activity and physic-chemical properties.

### Soil physicochemical and biological properties

The physicochemical properties of airdried soil were determined in up to three technical replicates, as previously stated in the study (Wu et al., 2013; Wang et al., 2021).The enzyme activities were measured after the plants were harvested. Soil invertase activity was study by colorimetric method using 3,5-dinitrosalicylic acid; soil peroxidase was analyzed by colorimetric method; the soil catalase activity was identified by potassium permanganate titration method; measurement of soil urease by the sodium-phenol colorimetric method; colorimetric determination of soil alkaline phosphatase by p-nitrophenyl disodium phosphate (Guan, 1986).

### Extraction and profiling of rhizosphere soil metabolites

The soil sample was collected in the stage of maturity of the plant and stored at -80°C. The extraction procedure of soil metabolite and detection was previously described by (Song et al., 2020). In brief, first add 2 g soil in 15 ml tube, and add 3 ml 75% methanol, 3 ml ethyl acetate, and 10 μl adonitol (10 mg/ml), oscillating 30 s. Then put the samples in shaker, 300 rpm, 4°C for 30 min, and then sonicate for 10 min. Afterwards, the samples were centrifuged at 12,000 rpm and 4°C for 10 min, and transferred the supernatants into a new tube. Repeat the step, and then all the supernatants were freeze drying. Then add 100 μl of methoxyamine hydrochloride into the samples, 37°C for 2 h. Final, add 70 μl silylating reagents (MSTFA) filled with samples, and reacted at 37°C for 30 min with continuous oscillating, then were centrifuged and the supernatant stored at -20°C for testing.

The samples were analyzed by GC-MS (TQ8040, Shimadzu, Japan). The extraction and detection of soil metabolites were previously described by Jiang et al. (2022). Briefly, the column oven temperature procedure was as follows: 90°C-170°C by 5°C/min, hold for 2 min, then 170°C-280°C by 10°C/min, then 280°C-300°C by 20°C/min, hold for 5 min. The samples were vaporized at 280°C by injection port. The flow linear velocity was 42.8 cm/s, the flow rate was set at 1.38 ml/min. The temperature of the ion source (EI) and interface were 200 and 250°C respectively, the solvent cut time was 3 min, the ionization energy was 70 eV.

The data were screened by NIST 2014. The compounds with more than 80% similarities were retained for the analysis. PCA-X and OPLS-DA for variance of root exudates between the different treatments was employed by using SIMCA 14.1.

### Extraction of soil genomic DNA

Each sample was weighed 0.7 g of soil, and use BioFast Soil Genomic DNA Extraction kit (BioFlux Hangzhou, China) to extracted the DNA. Then use the Nanodrop 2000C Spectrophotometer (Thermo Fisher Scientific, United States) to detection concentration. The bacterial community region (V3-V4) and the fungi region (ITS1) were used to sequenced. The samples were then commissioned to be sequenced by Biomarker Technologies (Beijing, China) using the Illumina Novaseq2500 platform.

A quantitative PCR assay was performed on the CFX96 Real-Time system (Bio-RDA, United States) to determine the abundance of microorganisms in different rhizosphere soils, including total soil bacteria, total fungi, Actinomycetes, Firmicutes, Bacteroides, and Acidobacteria. The bacterial primers were Eub338/Eub518; Fungi: ITS1F/ITS4; Actinobacteria: Actino235/Eub518; Firmicutes: Lgc353/Eub518; Bacteroidetes: Cfb319/Eub518; Acidobacteria Acid31/Eub 518 (**Supplementary Table 1**). The plasmid vectors with the specific fragment were constructed through gene cloning and vector link transformation in the early stage of the laboratory. Based on the optimized PCR system, fluorescence quantitative PCR was used for amplification. Four independent quantitative PCR assays were performed for each treatment.

### Exogenous addition of specific bacteria

Based on the previous screening in our laboratory, the specific bacteria (*Pseudomonas aeruginosa* 9, *Bacillus amyloliquefaciens* 4, *Bacillus subtilis* 35, *Bacillus subtilis* 74, *Bacillus halotolerans* 75) which isolated from the rhizosphere soil of *A. bidentata* (Wang et al., 2021), were used for the antagonistic experiments against the pathogenic fungi of *R. glutinosa* (*F. oxysporum* and *F. solani*) in lab as described by Wu et al. (2015). Then we added those specific bacteria to the pots under continuously planted *R. glutinosa*. Briefly, the specific bacteria were inoculated separately into 100 ml LB medium and incubated in shaker at 200 rpm and 37°C, when the OD_600_ value of the liquid reaches 0.8-1.0, centrifugate and remove the supernatant, then resuspend with 50 ml of distilled water, then add it to the potted treatments (3-gallon size bucket), at the same time, take and add the equal amounts of distilled water to blank pot as the control treatment. *R. glutinosa* was planted at the same density (2 plants/pot) on April 15 in 2021, the pot test was conducted on June 15th, 2021, repeat every 7 days, adding bacterial liquid four times in total. Each treatment had four replicates. The treated plants per pot were harvested on November 20^th^, 2021, then the fresh weight of the roots was measured and recorded. Then, a quantitative PCR assay used to determine the number of pathogenic fungi which belong to the *Fusarium* genus. The bacterial primers were ITS 1F/AFP308 (**Supplementary Table 1**).

### Statistical analyses

Apply Usearch (Edgar, 2013) to cluster reads with similarity greater than 97.0% to generate OTUs. Use SILVA as reference database for taxonomic annotation. Alpha diversity was analysed by QIIME2 (V1.9.1) (Bolyen et al., 2019). The more bioinformatics analysis is completed by using BMKCloud (http://www.biocloud.net/). The relationships between environmental factors and microbial abundances were explored by redundancy analyses (RDAs). All data were subjected to one-way analysis of variance (ANOVA) using the least significant difference (LSD, P<0.05) multiple range test in Data Processing System version (DPS) 7.05 software.

## Results

### Effects of intercropping on *Rehmannia glutinosa* growth

The *A. bidentata* and *R. glutinosa* yields are visualized in (**Figures 1 C–F**). Data pooted for two years (2020-21) showed that the effect of the intercropping systems on the growth of *A. bidentata* in these treatments was slight. However, the yield of *R. glutinosa* under consecutive monoculture cropping was significantly lower than that of the first crop (FR) yield. The yield of *R. glutinosa* proliferated in a nylon mesh barrier (SR) treatment by 31.63% and 35.76%, in the compares with those under no root barrier treatment (NR) and the completely root separated (MR) treatment. There existed no major difference in the yield of *R. glutinosa* in the NR and MR treatments. In comparison with the M treatment, the S treatment produced 1.17 LER (the average two-year land equivalent ratio) and the system productivity index (SPI) increased by 16.92%, while there was no significant difference in LER value between N and M treatments (**Supplementary Table 2**). Therefore, the results of this study suggest that S treatment with *A. bidentata plants* contributed to increase the yield of *R. glutinosa* under continuous cropping system.

### Soil physicochemical and biological properties

The physicochemical properties of the soils are shown in (**Table 1**, **Supplementary Table 3**). The pH of soil samples ranged from 7.14 to 7.58 and decreased significantly with increasing years of transplanting. Compared to FR rhizosphere soil, monoculture of *R. glutinosa* increased soil TN, TP, AN and AK contents, but decreased TK, AK and organic matter contents. AP and organic matter content in the rhizosphere soil were increased under SR and NR treatments in comparison to that in MR treatment. And AP in MA rhizosphere soil was higher than that under SA and NA treatments. Meanwhile, the activities of catalase, peroxidase, sucrose, and urease were higher in the rhizosphere soil of FR than those in MR regime, while the activities of alkaline phosphatase were lower. The MA rhizosphere soil had significantly higher activities of catalase, peroxidase, urease and sucrase than the MR. Activities of catalase, peroxidase, urease and sucrase were increased by 3.6%, 2.7%, 27.3% and 12.2%, respectively, in SR rhizosphere soil versus MR counterpart.

**Table 1:** Content of soil under different planting patterns.

Sample	TN(g/kg )	TK(g/kg )	TP(g/kg )	Available N (mg/kg)	Available K (mg/kg)	Available P(mg/kg)	Organic matter (g/kg)	pH
FR	2.31 ± 0.06 bc	0.92 ± 0.03 a	0.97 ± 0.01 d	194.00 ± 4.58 d	112.46 ± 1.91 c	315.26 ± 4.66 abc	30.58 ± 1.64 ab	7.48 a
MR	2.96 ± 0.55 a	0.73 ± 0.06 bc	1.21 ± 0.11 bc	256.67 ± 14.57 a	201.49 ± 6.77 a	276.90 ± 9.34 d	29.55 ± 0.46 b	7.15 a
MA	2.08 ± 0.41 c	0.72 ± 0.01 bc	1.04 ± 0.15 cd	186.67 ± 10.70 d	88.27 ± 2.49 de	325.46 ± 25.38 a	30.87 ± 0.94 ab	7.39 a
NR	2.75 ± 0.27 ab	0.73 ± 0.03 bc	1.25 ± 0.13 ab	228.67 ± 10.69 bc	214.60 ± 18.88 a	305.66 ± 9.03 abc	31.67 ± 2.15 a	7.14 a
NA	2.87 ± 0.27 a	0.57 ± 0.01 c	1.27 ± 0.13 ab	235.67 ± 7.23 b	102.40 ± 4.62 cd	316.83 ± 14.62 ab	30.20 ± 0.22 ab	7.33 a
SR	1.90 ± 0.14 c	0.59 ± 0.01 c	1.26 ± 0.11 ab	216.00 ± 6.56 c	172.59 ± 7.02 b	299.41 ± 16.36 bcd	31.79 ± 1.20 a	7.32 a
SA	1.96 ± 0.16 c	0.88 ± 0.05 ab	1.40 ± 0.12 a	184.33 ± 5.69 d	82.99 ± 6.20 e	291.01 ± 13.82 cd	30.77 ± 0.15 ab	7.21 a

FR, newly planted R. glutinosa rhizosphere soil; MR, plastic film barrier treatment R. glutinosa rhizosphere soil; MA, plastic film barrier treatment A.bidentata rhizosphere soil; SR, nylon mesh barrier treatment R. glutinosa rhizosphere soil; SA, nylon mesh barrier treatment A.bidentata rhizosphere soil; NR, no root barrier R. glutinosa rhizosphere soil; NA, no root barrier A.bidentata rhizosphere soil.

Different letters in columns show significant differences determined by LSD’s test (p<0.05, n=3).

### Impact of interspecific root compartment regime on the metabolite profiles of rhizosphere soil

**Figure 2:**
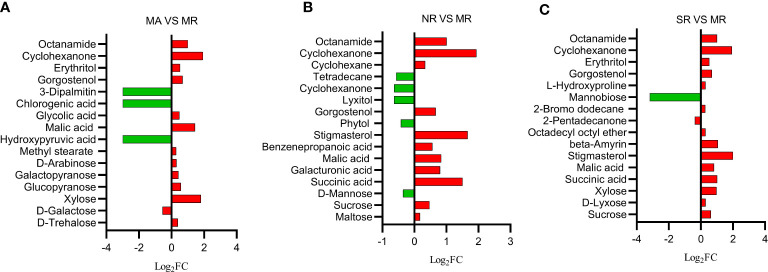
Histogram of differential soil compounds in comparison groups. The abscissa is log_2_FC of differential metabolites, and the ordinate is differential metabolites. **(A)** Red represents that MA is up-regulated than MR, and green represents down-regulated. **(B)** Red represents that NR is up-regulated compared to MR, and green represents down-regulated. **(C)** Red represents that SR is up-regulated than MR, and green represents down-regulated.

The nontargeted gas chromatography-mass spectrometry (GC-MS) metabolomic results revealed 141 metabolites of rhizosphere soils, and their functions were identified, including sugars (25), organic acids (38), esters (10), alcohols (21), lipids (15), and others (32). Among them, the main groups were sugars (18%) and organic acids (27%). The one-way ANOVA further revealed that 16 different soil metabolites were screened between MR rhizosphere soils and MA counterpart. These included six sugars such as D-alginate, D-galactose, xylose, etc. Five organic acids and their derivatives such as malic acid, glycolic acid and obscure esters and alcohols. As shown in **Figure 2A**, most of the metabolites were up-regulated under MA compared to those in MR. A total of 12 different metabolites were retrieved from the metabolites extracted from SR rhizosphere soil and MR counterpart (**Figure 2B**), including sugars, organic acids, esters and others involved in starch and sucrose metabolism, amino and nucleotide metabolism such as sucrose, xylose, succinic acid, malic acid, legumes sterols and others. All metabolites were up-regulated with respect to MR, except mannobiose or 2-pentadecanone. The majority of metabolites screened from the rhizosphere soils in NR and MR were organic acids and alcohols (**Figure 2C**), including succinic acid, malic acid, stigmasterol, phytol, and maltose. Nine metabolites were up-regulated in NR, while five were down-regulated when measured against metabolites in MR.

### Effect of different treatments on microbial alpha diversity of rhizosphere community

Among the 16S rRNA sequencing data, 1,200,852 pairs of reads were acquired from sequencing 15 samples, and a total of 1,196,302 clean tags were created after splicing and filtering of double ended reads. At least 79,565 clean tags were produced per sample, with an average of 79,753 clean tags generated. ITS high-throughput sequencing yielded 1,180,036 effective clean tags in the 15 soil samples, respectively. At least 66,386 clean tags were generated for each sample, and an average of 78,669 Clean tags was generated. The sequences from all of the samples clustered into 6,140 fungal OTUs and 23,746 bacterial OTUs based on an identity threshold of 97%, (**Supplementary Tables 4**, **5**). The Shannon and Simpson indices of the fungi from the FR and SR rhizosphere soils were higher than those from the MA, MR and NR rhizosphere soils. The Chao1 and ACE indices showed only a slight difference in these treatments. The Chao1 indices of the bacterial communities in NR were higher than those in the other regimes. The Shannon diversity index of NR and SR was greater than that of MR.

### Beta diversity of rhizosphere microbial community under different treatments

**Figure 3:**
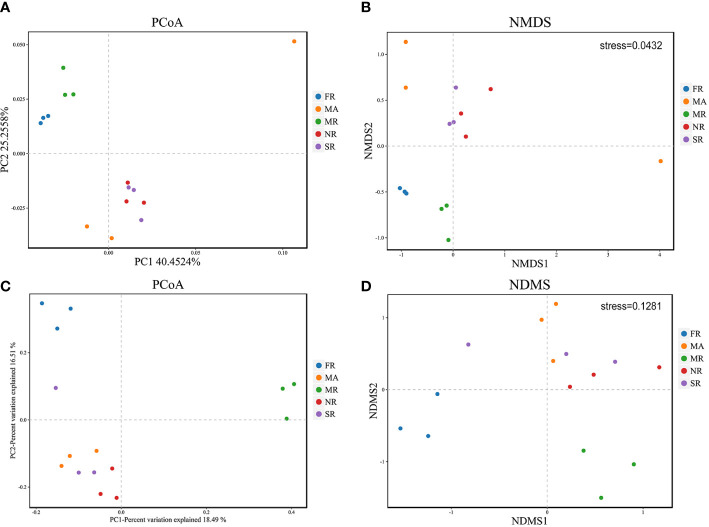
Principal Coordinate Analysis (PCoA) and of 16S rRNA and ITs diversity in the rhizosphere of the five samples **(A, C)**. Variations of bacterial and fungal communities among different samples based on Nonmetric Multidimensional Scaling (NMDS) analysis **(B, D)**.

PCoA shows general structural resemblance of the bacterial and fungal community structure across samples using the OTUs of 16S rRNA sequencing data and ITS rRNA sequencing data. For bacteria, PCo1 accounted for 40.45%, while PCo2 constituted only 25.26% of the variation detected in the bacterial community composition (**Figure 3A**). However, for fungi, PCo1 represented 18.49%, while PCo2 accounted for 16.51% of the variation detected in the composition of the fungal community (**Figure 3C**). Analysis of NMDS at the OTU levels variation in fungal and bacterial beta diversity according to Euclidean distance dissimilarities (**Figures 3B, D**). Bacterial and fungal communities were clustered in the rhizosphere soils of FR and MR, as well as in the soils of SR and NR. In addition to this, the fungal and bacterial communities in the rhizosphere soil of *A. bidentata* under MA were separated from those in the rhizosphere soil of *R. glutinosa* in each of the treatments.

### Shifts in microbial community structure in response to different cropping systems

We identified 26 bacterial phyla across all of the samples. The dominant phyla were Proteobacteria (29.25%-34.51%), Gemmatimonadetes, Acidobacteria (26.88%-32.35%), Actinobacteria, Chloroflexi, Firmicutes, Rokubacteria, Bacteroidetes, Nitrospirae (total relative abundance > 95%). Proteobacteria under the MA regime were detected at a higher relative abundance than other phyla, while Actinobacteria exhibited the opposite trend. Furthermore, the relative abundances of Acidobacteria under MR more significantly decreased than that of FR. The relative abundances of Firmicutes, Bacteroidetes, and Nitrospirae were increased in intercropping when compared with MR.

Taxonomic analysis of fungal sequences obtained 12 phyla from the valid sequence at a 97% similarity level. Based on the 10 most abundant bacterial phyla, the relative abundance of the dominant fungal phyla in the soil was similar among all the treatments, including Ascomycota (59.09%-64.25%), Basidiomycota (15.71%-29.96%), Mortierellomycota (2.41%-6.43%).

**Figure 4:**
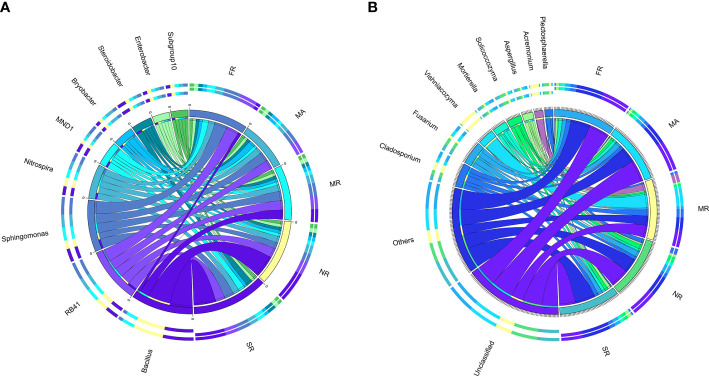
Distribution of dominant genera (top 10) in five different samples. **(A)** represents the bacterial community in the soil of the rhizosphere. **(B)** represented the fungal community in rhizosphere soil) The thickness of each ribbon represents the abundance of each taxon. The relative tick above the outer segment stands for the relative abundance of each taxon. Data were visualized using Circos (version 0.69, http://circos.ca/).

We explored differences in the relative abundances of the bacteria genera among different treatments. Except for unknown genera, *Bacillus*, *RB41*, *Sphingomonas*, *Nitrospira*, *MND1*, *Bryobacter*, *Steroidobacter*, *Enterobacter*, and *Subgroup-10* were dominant genera (**Figure 4A**). In MR, a significant decrease in relative abundances of *RB41*, *Sphingomonas, Nitrogenspira* and *MND1* as compared to those in FR. Compared with those in MR, the abundances of *Bacillus* and *Sphingomonas* in SR and NR had been remarkably improved by 73% and 69%. Meanwhile, *Nitrospira*, *Steroidobacter* in SR increased by 12.64%, 12.62% and 74.1%, and *Nitrospira*, *Bryobacter*, *Steroidobacter* in NR increased by 3.88%, 19.42%, and 26.97%, respectively.

The structure of the fungal community differed substantially among samples. Hierarchical cluster heat maps were used to analyze the 20 most abundant fungal genera in all samples (**Figure 4B**). The dominant genera across the samples under MR were *Vishniacozyma* (23.36%), *Acremonium* (11.20%), *Cladosporium* (10.56%) and *Fusarium* (3.61%). Furthermore, a higher abundance of Vishniacozyma under MR than others. In addition, SR significantly increased the relative abundances of *Mortierella*, *Aspergillus*, *Botryotrichum* and increased the relative abundances of *Vishniacozyma*, *Acremonium*, *Gibellulopsis, and Thanatephorus* compared to those in MR.

**Figure 5:**
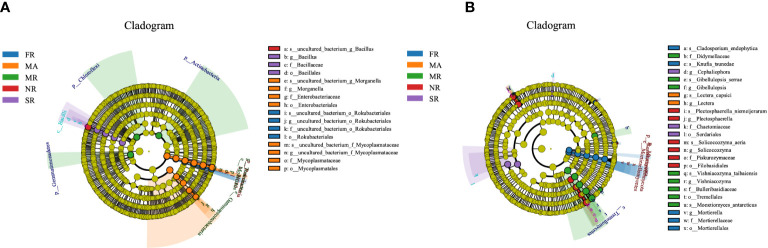
Bacterial and fungal under different planting patterns. (**A** means bacterial, **B** means fungal.) In the figure, circles from inner to outer layers represent the taxonomic level from phylum to species. The dots on circles represent a term on corresponding taxonomic level. The size of the dots indicates relative abundance. Coloring: Species with no significant difference are colored yellow. Other colors stand for different groups. Species with certain color means the abundance of this species is the highest in the corresponding group, which helps visualize the most important microbial communities in each group.

LEfSe analysis was adopted to detect differentially abundant bacteria and fungi in the rhizosphere soil under different treatments. The LEfSe analysis of the soil bacterial community showed 23 distinctly abundant taxa among the five groups (**Figure 5A**). Of the 23 taxa, the Bacillus gen*us* was enriched in soil from the rhizosphere under the treatment of SR and NR compared to the others. The enriched taxa in MA were the classes of the phylum Bacteroidetes, Gemmatimonadetes, and Bacteroidia. The distinctly abundant taxa in MR were the Actinobacteria, Chloroflexi Gemmatimonadetes and the phylum Rhizobiales. The Rokubacteria phyla, and the order Rokubacteriales were enriched in FR. In addition, a total of 27 abundant fungal taxa significantly differed across FR, MR, NR, SR and MA (**Figure 5B**). Among the 27 fungal taxa, 7 taxa were enriched in FR, mainly the Mortierellates order and the *Mortierella* genus. The abundant fungal taxa in MR were the Tremellales order. While the most distinctly abundant fungal taxa were *Cephaliophora* genus in the SR. The enriched fungal taxa in NR were the Filobasidiales order and the *Plectosphaerella* and *Solicoccozyma* genus.

Quantitative PCR analysis showed that FR and NR had the highest number of bacteria in the rhizosphere soil. However, the total amount of fungi in MA and NR rhizosphere increased than the others. The abundance of Acidobacteria phylum in MR rhizosphere soils was lower than other treatments. Intercropping significantly increased the relative abundances of Firmicutes phylum in SR rhizosphere soils and Actinomycetes phylum in NR rhizosphere soils (**Supplementary Figures 3**, **4**).

### Co-occurrence network and correlation analysis

**Figure 6:**
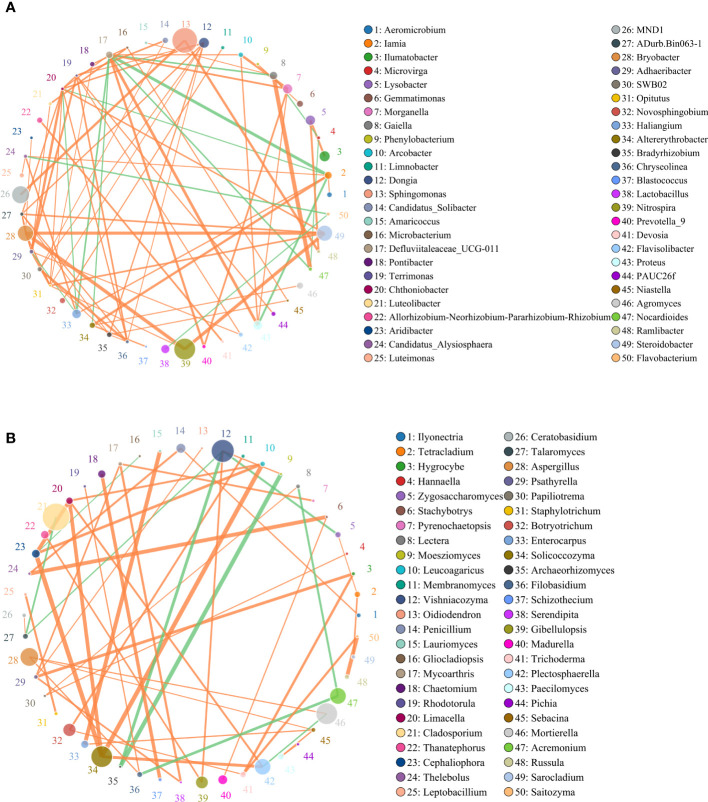
Circles represents species, size of circle represents the abundance; **(A)** means the results of bacterial and **(B)** means the results of fungal. The edges represent the correlation between the two species, the thickness of the edge represents the strength of the correlation, and the color of the line: orange represents the positive correlation, while green represents the negative correlation.

Spearman correlation analysis identified significant correlations between microbial taxa across all treatments. To compare the complexities of microbiome associations among all treatment soils, two networks were constructed based on the microbiomes identified in the 15 soil samples. Of those nodes and edges, soil bacterial relationships were more complicated than soil fungal relationships (**Figures 6A, B**). *Sphingomonas*, *MND1*, *Nitrospira*, *Steroidobacter*, *Bryobacter*, *Morganella*, *Haliangium* were widely distributed among bacteria genera, respectively (**Figure 6A**). Most of the bacteria genera had positive correlations among the 50 most abundant genera in the soil microbial community, including *Sphingomonas* and *MND1*, *Gaiella* and *Nocardioides*, *Steroidobacter* and *Nitrospira, and Bryobacter*. While *Haliangium* and *Steroidobacter*, *Defluviitaleaceae* and *Iamia*, *Nocardioides* were on negative correlations. The relatively high abundance of fungal communities was *Cladosporium*, *Vishniacozyma*, *Acremonium*, *Solicoccozyma*, *Morganella*, *Aspergillus*, etc., respectively (**Figure 6B**). Additional fungal genera were observed to have a positive correlation with each other, while only nine fungal genera were in the contrary.

**Figure 7:**
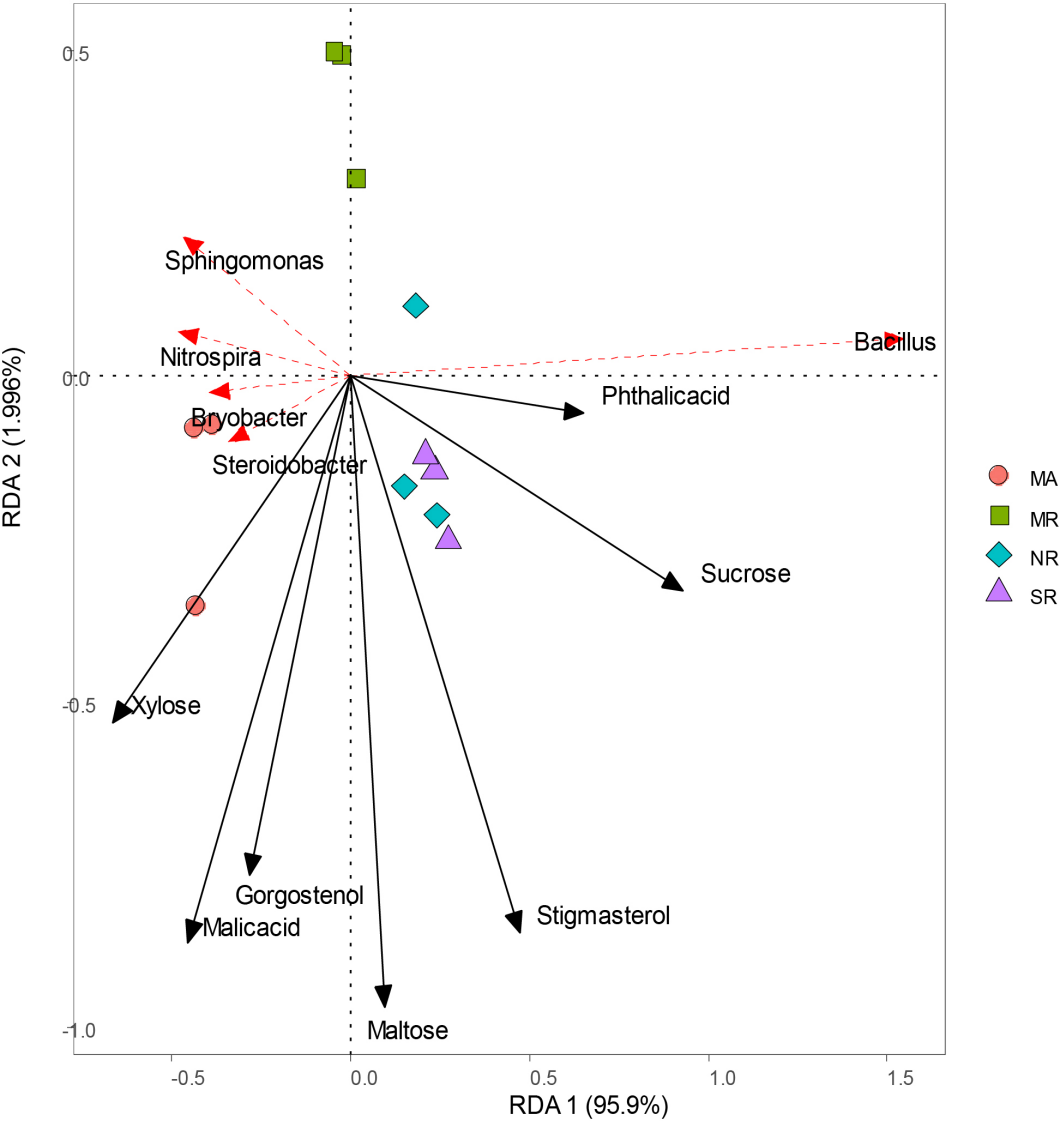
Redundancy analysis of soil microorganisms (genus) and main differential metabolites under different intercropping modes.

RDA analyses indicated that bacterial community structure changed substantially among all soils (**Figure 7**). Sucrose, stigmasterol, and phthalic acid levels were positively correlated with higher relative abundances of *Bacillus*, reverse was true in the case of xylose levels. Strong associations were found between malic acid and gorgostenol levels and the relative abundances of *Steroidobacter* and *Bryobacter*.

### Functional verification of key microorganisms *in vitro* interaction

**Figure 8:**
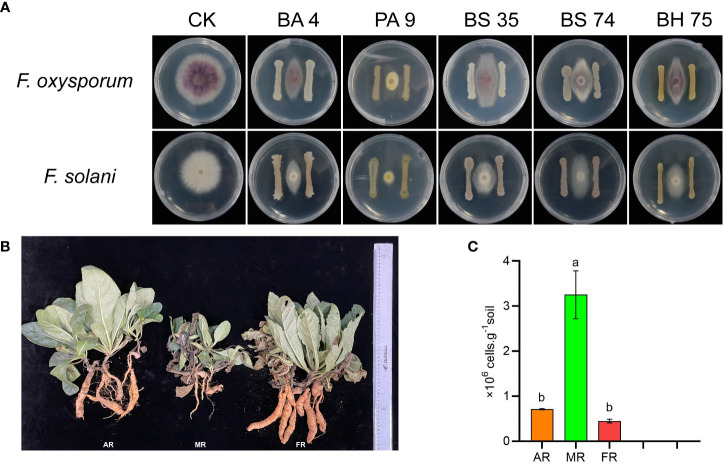
Functional verification of species microbe *in vitro* interaction. **(A)** means effect of different bacteria against the pathogenic fungi of *R. glutinosa*. PA 9 means *Pseudomonas aeruginosa* 9, BA 4 means *Bacillus amyloliquefaciens* 4, BS 35 means *Bacillus subtilis* 35, BS 74 means *Bacillus subtilis* 74, BS 75 means *Bacillus halotolerans* 75. **(B)** means growth of *R. glutinosa* under different treatments. AR means add the mix bacterial agent *R. glutinosa*, MR means monoculture *R. glutinosa*, and FR means first stubble *R. glutinosa.* The number of *Fusarium* in rhizosphere soil under each treatment **(C)**. Different letters in columns show significant differences determined by LSD’s test (p<0.05, n=3).

In this study, we found that the *B. amyloliquefaciens* 4, *B. subtilis* 74, *B. halotolerans* 75, and *Pseudomonas* 9 exhibited a strong antagonism against the two pathogenic fungi of *R. glutinosa* (**Figure 8A**). The root fresh weight of *R. glutinosa* plants significantly increased under the mixed bacterial liquid treatment (**Figure 8B**). In addition, qPCR results showed that exogenous addition of the bacterial solution remarkably reduced the pathogenic fungi in the rhizosphere soil of *R. glutinosa* (**Figure 8C**).

## Discussion

### Intercropping increases root biomass of *R. glutinosa*, induced changes in root exudate metabolites

In the multi-cropping system, intra- and interspecific competition among crop individuals is essential for the development and production of components. Compared with the competition in aboveground parts, root competition is also a key factor affecting crop growth. In the present study, the yield of *A. bidentata* showed no significant variation among the three treatments. Meanwhile, we found that the yield of *R. glutinosa* was higher in the S (a nylon mesh barrier) treatment than under the N (no root barrier) and M (plastic film barrier) treatments, while the yield of *R. glutinosa* was almost the same under the N and M treatments. Therefore, the results of this study suggest that intercropping with *A. bidentata* has an interspecific promotion effect on the growth of *R. glutinosa*. However, there was a competitive relationship between below ground of the two interspecific plants. In the presence of the simultaneous subterranean communication, the yield of *R. glutinosa* increased under the S treatment, showing the beneficial of intercropping, whereas the yield of *R. glutinosa* did not reflect the advantage of intercropping under the N treatment. We presume that this is attributed to the limited space under the pot experiment. The roots of *A. bidentata* occupied the space of *R. glutinosa*, thus creating a competitive relationship, which in turn affected the yield of *R. glutinosa*. This result further suggests that interactions between rhizosphere microorganisms and exudates under subterranean intercropping conditions may be the crucial for alleviating the replanting disease in *R. glutinosa*.

The regulation and alteration of rhizosphere microorganisms are mainly driven by root exudates. Based on the results of our experiments, the root exudate profiles varied between treatments; with the main differences focusing on malic acid, sugars and sterols. Our study indicated that malic acid, sugars, and sterols were positive with beneficial microbes. Sugar serves as primary energy sources for microbial growth in the rhizosphere. Our previous studies have shown that beneficial soil bacterial species (*Bacillus subtilis, Rhizobium leguminosarum*, and et al.) use various sugars and show chemotaxis, and the sugar exudates were reduced in response to pathogen inoculation (Abu-Nada et al., 2007; Chaparro et al., 2013; Wu et al., 2020). Sucrose has been mentioned as an essential element in the evolution of symbiotic plant-microbial interactions and potential plant defensive mechanisms (Vargas et al., 2009; Zhalnina et al., 2018). Other studies have shown that malic acid could recruit beneficial rhizosphere microorganisms. For example, Lakshmanan et al. (2012) found that inoculation of Arabidopsis leaves with pathogen resulted in the induction of malic acid secretion into the rhizosphere, which in turn enriched more beneficial rhizobacteria *Bacillus subtilis*. Likewise, watermelon roots could excrete malic acid to attract root colonization of the plant growth-promoting rhizobacterial *Paenibacillus polymyxa* (Ling et al., 2011). In our study, the relative abundance of *Bacillus* has increased in rhizosphere soil of the *R. glutinosa* plants in the intercropping systems. Therefore, our results suggest that intercropping changes the rhizosphere compounds of *R. glutinosa*, thereby enhanced the diversity and abundance of microorganisms in *R. glutinosa* rhizosphere, which indirectly improved the yield of *R. glutinosa*.

### Intercropping improved soil enzyme activity and rhizosphere microenvironment

Intercropping has repeatedly been demonstrated numerous occasions to improve ecosystem functioning, such as increased productivity and robustness, enhanced utilization efficiency of resources, and minimized environmental costs (Yu et al., 2022). However, in our study, the nutrient elements were improved in rhizosphere soil under each treatment caused by fertilization. Indeed, our previous study has shown that the available soil nutrients did not reduce in the case of consecutive monoculture of *R. glutinosa* and *R. pseudostellariae*, fertilization was not found to be active in combating replant disease (Wu et al., 2015a; Wu et al., 2019). Therefore, our results indicated that in the *A. bidentata*-*R. glutinosa* intercropping system, nutrient elements are not the key cause in alleviating the replanting disease of *R. glutinosa.* Moreover, relative to monocultures, intercropping may enhance soil fertility by raising soil organic matter (Li et al., 2021). In our study, soil fertility *via* observed increases in soil organic matter when comparing SR and NR with MR soils, this result confirmed the finding documented by previous research.

Soil fertility involves nutrient elements and soil enzymes. Previous studies have shown that suitable intercropping can enhance soil enzyme activity. As an example, soil invertase, urease, and alkaline phosphatase activities were increased under garlic-cucumber intercropping system when compared to monoculture, and the urease and alkaline phosphatase promotion by intercropped garlic were sustained up to garlic harvest (Xiao et al., 2012). Sorghum and cowpea intercropping improved the activity of acid and alkaline phosphatase in cowpea rhizosphere soil, resulted in a clear improvement P nutrition in cowpea, vigorous plant growth (Makoi et al., 2010). During our study, the catalase, peroxidase, urease and sucrase activity in continuous cropping *R. glutinosa* rhizosphere soil decreased compared to the first cropping. However, the activity of soil urease and invertase was encouraged under S and N compared with that in M regime. These results showed that in the intercropping system, the allelopathy mediated by root exudates improved organic matter and increased the activities of invertase and urease activities in *R. glutinosa* rhizosphere soil, alleviated the replanting disease of *R. glutinosa*.

### Intercropping enriches beneficial microorganisms to antagonistically act against the pathogen infection

Replanting disease is a classical negative soil memory that occurs in the context of intensive consecutive monoculture. For exemplified by the fact, those plants, such as potatoes, watermelons, tobacco, *R. pseudostellariae*, and *P. notoginseng*, will form a serious replant disease under monoculture. Replanting disease typically results in an increase in the number of pathogens and reduction in the level of beneficial microbe (Dong et al., 2018; Liu et al., 2021). Former research has suggested that some species of *Mortierella*, which generate antibiotics, as well as several isolates, have been shown to be potential antagonistic agents against a range of plant pathogens (Wang et al., 2021). LEfSe results showed a significant decrease in the populations of *Mortierella* in the rhizosphere soil as the number of monoculture years increased. Furthermore, our previous research indicated that *R. glutinosa* monoculture caused an increased *F. oxysporum* and a decreased abundance of beneficial bacterial microbes (i.e., *Pseudomonas* spp.*, Bacillus* spp.) (Wu et al., 2015; Wu et al., 2018a; Wu et al., 2018b). Earlier studies have revealed that biofertilizer amendment could mitigate the replanting disease of medicinal plants by modifying the rhizosphere microbial colony and inhibiting the abundance of pathogenic *Fusarium*, *Ilyonectria*, and *F. oxysporum* (Wu et al., 2021). From here, we observed that, compared to MR treatment, SR and NR treatments increased the relative abundance of potentially beneficial microbes at the genus level of *Nitrospira*, *Steroidobacter* and *Bacillus*. The result has demonstrated that the genus *Steroidobacter* serves as a bacterial antagonist to phytopathogenic fungi, promoting plant growth (White et al., 1996). The genus *Nitrospira* is a worldwide community of nitrite oxidizers and a critical component of nitrogen-cycling microbial assemblages (Daims et al., 2015). Generally, *Bacillus* spp. have a wide variety of activity antagonistic to plant pathogenic bacteria, fungi and viruses (Fira et al., 2018). Du et al. (2020) revealed that the DU-1 belongs to the genus *Bacillus* and showed an antagonistic effect against *F. oxysporum.* Therefore, our results showed that intercropping increased the relative abundance of beneficial bacteria in the *R. glutinosa* rhizosphere. In the current study, it was confirmed by plate standoff experiments that *Bacillus* spp. isolated from the soil had a significant antagonistic effect on the specific pathogenic pathogens (*F. oxysporum* and *F. solani*) of *R. glutinosa*. Moreover, exogenous additive experiments have shown that these beneficial bacteria exhibit relatively strong biological activities, consequently enhanced the yield of *R. glutinosa*. However, the *Fusarium* was still the dominant genus in all the samples. Those results suggest that intercropping did not directly lead to the decrease of pathogens in *R. glutinosa* rhizosphere but by increasing the abundance of beneficial bacteria to compete for the ecological niche and reduce the damage of pathogens to host plants. Our findings are in agreement with a similar study (Wu et al., 2020), which indicated that the intercropped *R. pseudostellariae* plants can enhance rhizosphere microorganisms biodiversity and stabilize selection pressure on *Fusarium*, thus preventing the capability of the pathogenic fungus to undergo rapid evolution towards higher virulence. In addition, a variety of root secretions (analogue or antibiont) derived in the course of intercropping might act as allelochemicals to remediate the unbalance rhizosphere ecosystem. In view of the above, these findings suggest that intercropping *A. bidentata* can ameliorate soil biological properties and mitigate the monocropping obstacle of *R. glutinosa*.

## Conclusion

In summary, our study suggested that *R. glutinosa*-*A. bidentata* intercropping systems can alleviate the consecutive monoculture problem of *R. glutinosa*. In this study, the mechanism by which intercropping alleviated the consecutive monoculture problem was that species diversity caused a change in root exudates, elevated the abundance of PGPR, activated soil enzyme activity, and repaired the imbalanced microorganisms. However, interspecific competition exists between *R. glutinosa* and *A. bidentata*, which is linked to the distance of the roots. In field experiments, how strengthening the root interaction and reduce physical competition is also urgent problem to be solved. In the long term, we need to explore the formation of soil memory mechanism, which is an important theoretical basis for solving the consecutive monoculture problem, and has a positive effect on agriculture sustainability.

## Data availability statement

The data presented in the study are deposited in the NCBI repository, accession number PRJNA878926 (https://www.ncbi.nlm.nih.gov/bioproject/PRJNA878926).

## Author contributions

CF and LW conceived and directed the project. YZL, YL, CZ, and JW did all of experiments. YZL, LW and TC performed the integrated data analysis. YZL and WL wrote the manuscript. YJ and WN provided linguistic and scientific assistance. All authors contributed to the article and approved the submitted version.

## Funding

This work was supported by grants from the National Natural Science Foundation of China (81973412, 81573530), the Natural Science Foundation of Fujian Province (2021J01090), and Science and Technology Innovation Fund of Fujian Agriculture and Forestry University (CXZX2020039A).

## Conflict of interest

The authors declare that the research was conducted in the absence of any commercial or financial relationships that could be construed as a potential conflict of interest.

## Publisher’s note

All claims expressed in this article are solely those of the authors and do not necessarily represent those of their affiliated organizations, or those of the publisher, the editors and the reviewers. Any product that may be evaluated in this article, or claim that may be made by its manufacturer, is not guaranteed or endorsed by the publisher.
