# Supplementary material for: Intercropping with Achyranthes bidentata alleviates Rehmannia glutinosa consecutive monoculture problem by reestablishing rhizosphere microenvironment
Source: Front Plant Sci. 2022 Nov 22;13:1041561. doi: 10.3389/fpls.2022.1041561 (PMC9724704; doi:10.3389/fpls.2022.1041561)
Supplement: Supplementary file 1 [file DataSheet_1.docx]

Intercropping with *Achyranthes bidentata* alleviates *Rehmannia glutinosa* consecutive monoculture problem by reestablishing rhizosphere microenvironment

Yazhou Liu^1,2^†, Ye Liu^1,2^†, Chunli Zeng^1,2^, Juanying Wang^3^, Witness Joseph Nyimbo^1,2^, Yanyang Jiao^1,2^, Linkun Wu^1,2^, Ting Chen^1,2^, Changxun Fang^1,2*^, Wenxiong Lin^1,2*^

^1^Fujian Provincial Key Laboratory of Agroecological Processing and Safety Monitoring, College of Life Sciences, Fujian Agriculture and Forestry University Fuzhou, 350002, Fujian, China.

^2^ Key Laboratory of Crop Ecology and Molecular Physiology (Fujian Agriculture and Forestry University), Fujian Agriculture and Forestry University, Fuzhou, 350002, China.

^3^Key Laboratory of Plant Resources Conservation and Germplasm Innovation in Mountainous Region (Ministry of Education), Guizhou Key Lab of Agro-bioengineering, Institute of Agro-bioengineering/College of life science, Guizhou University, Guiyang, 550025, Guizhou Province, China.

† Both authors contributed equally to this paper

* **Corresponding author:** Changxun Fang or Wenxiong Lin

Fujian Agriculture and Forestry University,

Fuzhou 35002, Fujian, China

Phone: +86-0591-83737535;

Fax: +86-0591-83769440

E-mail: [changfangxingyx@163.com](mailto:changfangxingyx@163.com), [lwx@fafu.edu.cn](mailto:lwx@fafu.edu.cn)


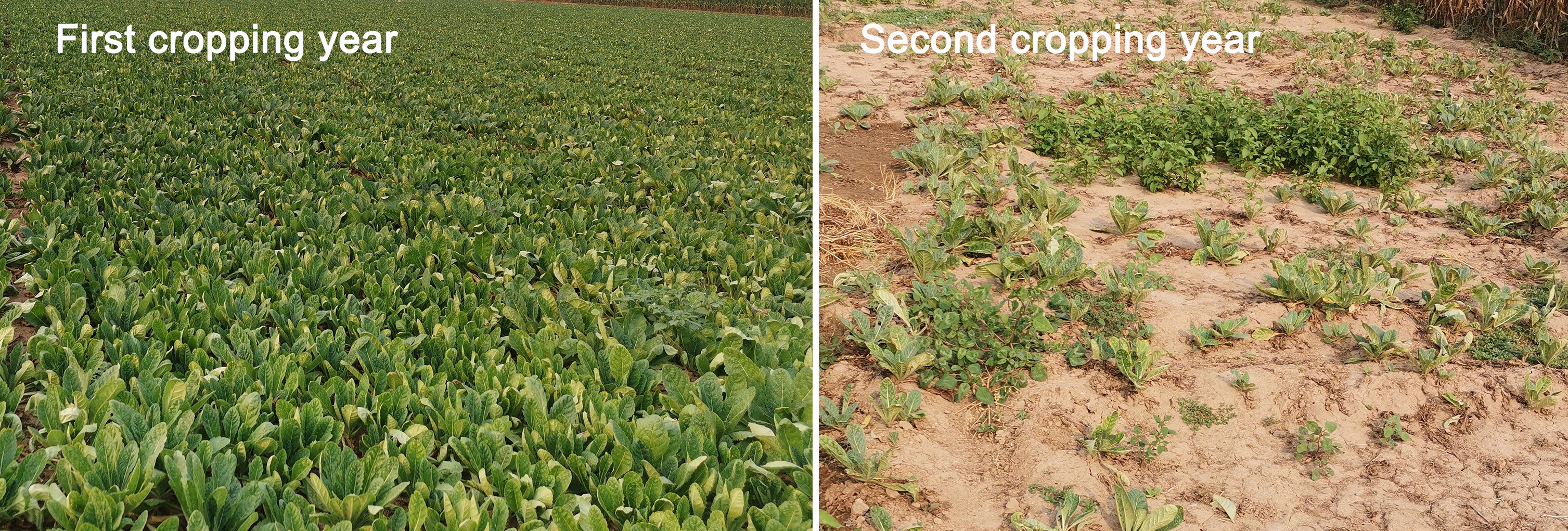


Figure S1: Field phenotypes of *Rehmannia glutinosa* under consecutive monoculture


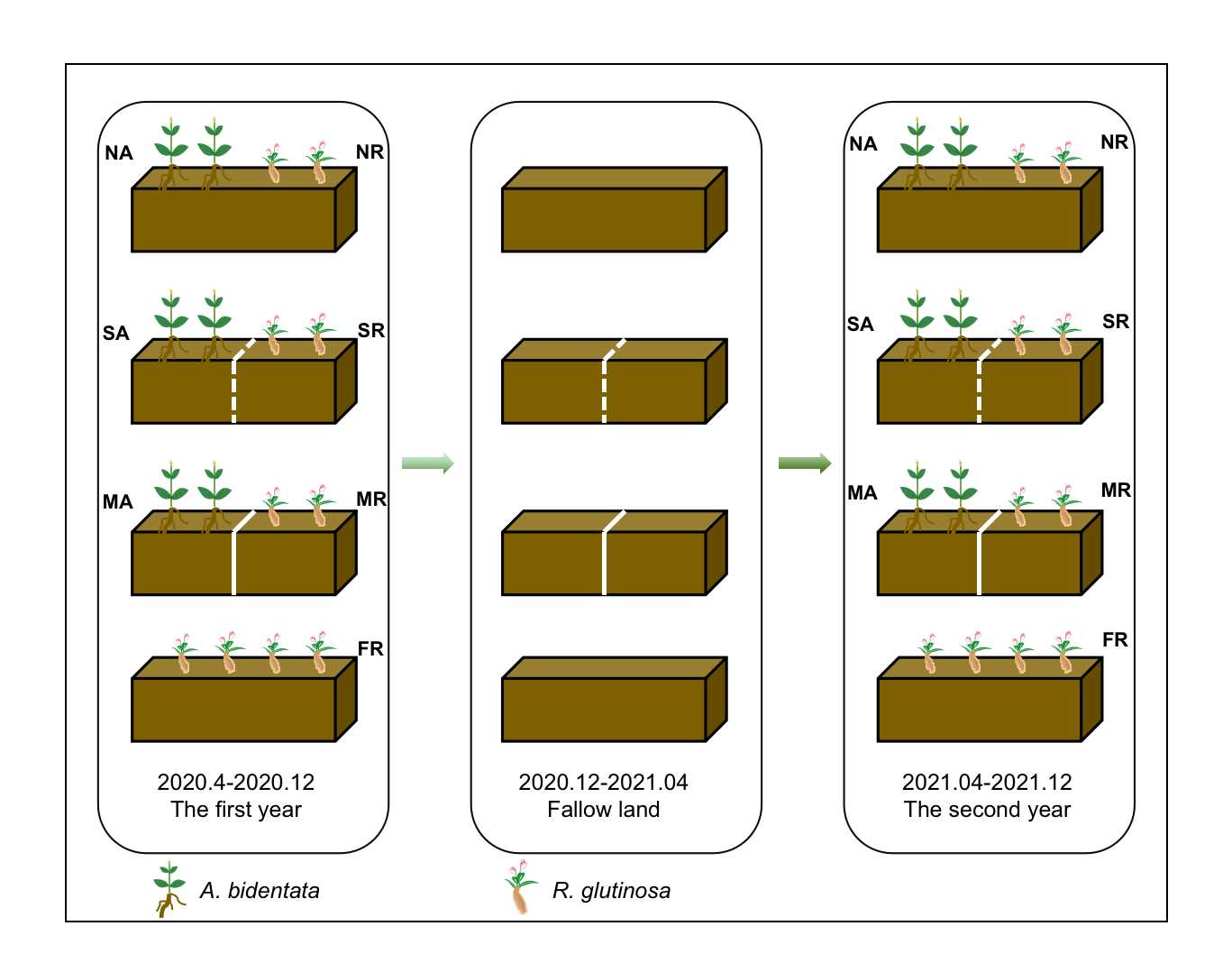


Figure S2: Phenotypic distribution of yield traits

Table S1 The primers and parameters for qPCR

| Name | Sequence | Reactions conditions |
| --- | --- | --- |
| Eub338 | ACT CCT ACG GGA GGC AGC AG | 95℃20s,53℃20s,72℃20s |
| Eub518 | ATT ACC GCG GCT GCT GG |  |
| ITS1F | CTT GGT CAT TTA GAG GAA GTA A | 95 ℃45s,51 ℃45s,72 ℃60s |
| ITS4 | TCC TCC GCT TAT TGA TAT GC |  |
| Lgc353 | GCA GTA GGG AAT CTT CCG | 95 ℃60s,60 ℃30s,72 ℃60s |
| Eub518 | ATT ACC GCG GCT GCT GG |  |
| Actino235 | CGC GGC CTA TCA GCT TGT TG | 95 ℃60s,60 ℃30s,72 ℃60s |
| Eub518 | ATT ACC GCG GCT GCT GG |  |
| Cfb319 | GTA CTG AGA CAC GGA CCA | 95 ℃45s,65 ℃30s,72 ℃45s |
| Eub518 | ATT ACC GCG GCT GCT GG |  |
| Acid31 | GAT CCT GGC TCA GAA TC | 95 ℃50s,50 ℃30s,72 ℃60s |
| Eub518 | ATT ACC GCG GCT GCT GG |  |
| ITS 1F | CTT GGT CAT TTA GAG GAA GTA A | 95℃50s,60℃45s,72℃60s |
| AFP308 | GGA ATT AAC GCG AGT CCC AA |  |

Table S2 Effects of different planting patterns on LER and SPI

|  | LER | | | SPI | | |
| --- | --- | --- | --- | --- | --- | --- |
| year | M | S | N | M | S | N |
| 2020 | 1 | 1.16 | 0.96 | 38.79 | 45.01 | 37.24 |
| 2021 | 1 | 1.18 | 1.02 | 31.91 | 37.66 | 32.66 |
| average | 1 | 1.17 | 0.99 | 35.35 | 41.33 | 34.95 |

Table S3 Soil enzyme activity under different cropping patterns

| Sample | Catalase (umol/h/g) | Peroxidase (nmol/h/g) | Urease（mg/g) | Invertase（mg/d/g) | Alkaline phosphatase（umol/h/g) |
| --- | --- | --- | --- | --- | --- |
| FR | 254.38±0.20d | 952.24±13.19a | 10.98±0.10a | 18.48±1.16ab | 0.31±0.03c |
| MR | 248.39±1.29e | 633.16±69.07c | 8.71±0.01be | 17.23±0.09c | 0.63±0.11b |
| NR | 236.98±0.31f | 499.24±20.74e | 10.54±0.05b | 17.45±0.04bc | 0.73±0.06b |
| SR | 257.17±0.94c | 650.4±71.23c | 11.09±0.18a | 19.25±0.13a | 0.66±0.04b |
| MA | 261.72±0.30b | 767.15±41.93b | 9.26±0.08d | 18.90±0.05a | 0.71±0.01b |
| NA | 254.19±1.44d | 606.74±18.84cd | 9.84±0.21c | 17.40±0.11bc | 0.95±0.16a |
| SA | 264.05±0.40a | 530.53±56.58de | 10.95±0.10a | 19.43±0.08a | 0.66±0.03b |

FR: newly planted *R. glutinosa* rhizosphere soil; MR: plastic film barrier treatment *R. glutinosa* rhizosphere soil; MA: plastic film barrier treatment *A. bidentata* rhizosphere soil; SR: nylon mesh barrier treatment *R. glutinosa* rhizosphere soil; SA: nylon mesh barrier treatment *A. bidentata* rhizosphere soil; NR: no root barrier *R. glutinosa* rhizosphere soil; NA: no root barrier *A. bidentata* rhizosphere soil.

Table S4 Diversity index of bacterial community in rhizosphere soil under different planting patterns

| Bacteria | Number  of OTUs | ace | Simps-on | chao1 | Shannon | Coverage (%) |
| --- | --- | --- | --- | --- | --- | --- |
| FR | 1555.33±4.70 ab | 1584.10±6.53 ab | 0.99 a | 1597.21±10.6 ab | 9.11±0.01 a | 99.84 |
| MA | 1538.33±22.18 b | 1568.38±11.44 b | 0.99 a | 1575.71±10.21 b | 8.60±0.38 a | 99.83 |
| MR | 1556.67±3.84 ab | 1584.61±1.74 ab | 0.99 a | 1597.53±3.5 ab | 9.03±0.03 a | 99.83 |
| NR | 1591.00±2.52 a | 1607.56±1.99 a | 0.99 a | 1621.43±2.68 a | 8.91±0.05 a | 99.9 |
| SR | 1584.00±0.00 ab | 1598.49±0.11 a | 0.99 a | 1607.31±0.32 ab | 8.90±0.04 a | 99.91 |

FR: newly planted *R. glutinosa* rhizosphere soil; MR: plastic film barrier treatment *R. glutinosa* rhizosphere soil; MA: plastic film barrier treatment *A. bidentata* rhizosphere soil; SR: nylon mesh barrier treatment *R. glutinosa* rhizosphere soil; NR: no root barrier *R. glutinosa* rhizosphere soil; Different letters in columns show significant differences determined by Tukey’s test (p≤0.05, n = 3).

| Fungi | Number  of OTUs | ace | Simpson | chao1 | Shannon | Coverage (%) |
| --- | --- | --- | --- | --- | --- | --- |
| FR | 494.67±18.92 a | 879.99±68.46 a | 0.98±0.01 a | 657.10±42.14 a | 7.30±0.48 a | 99.88 |
| MA | 361.67±22.32 c | 1003.58±70.55 a | 0.96±0.01 ab | 633.86±51.89 a | 6.37±0.39 ab | 99.88 |
| MR | 354.33±17.94 c | 899.14±52.20 a | 0.91±0.04 b | 591.11±33.12 a | 5.24±0.41 b | 99.87 |
| NR | 396.00±10.15 bc | 901.10±33.04 a | 0.96±0.03 ab | 628.07±15.60 a | 6.05±0.74 ab | 99.83 |
| SR | 440.00±21.80 ab | 957.91±33.88 a | 0.97±0.004 a | 655.71±32.54 a | 6.79±0.45 a | 99.86 |

Table S5 Diversity index of fungal community rhizosphere soil under different planting patterns


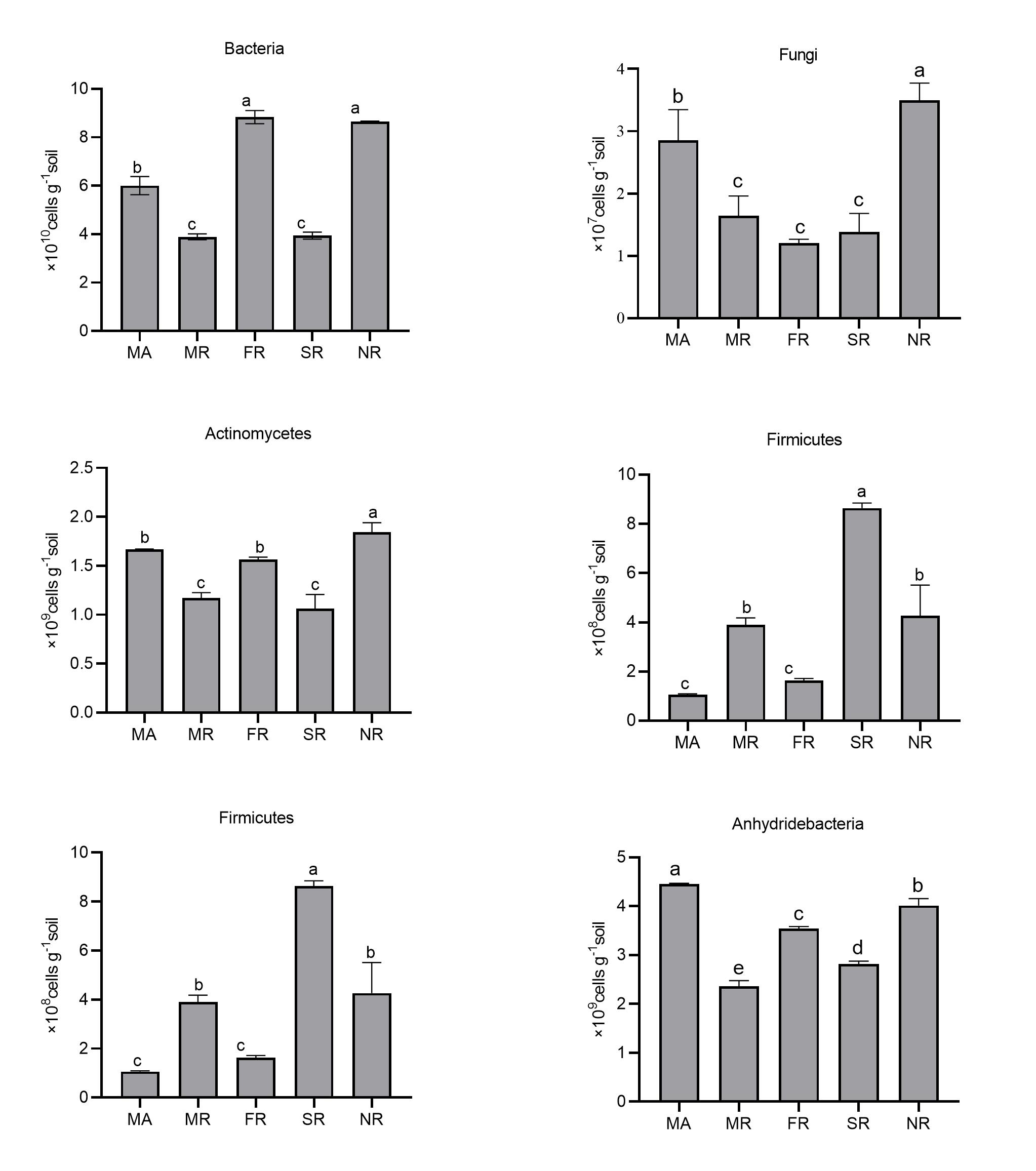


Figure S3 qPCR results of main microbial communities in rhizosphere soil under different planting patterns


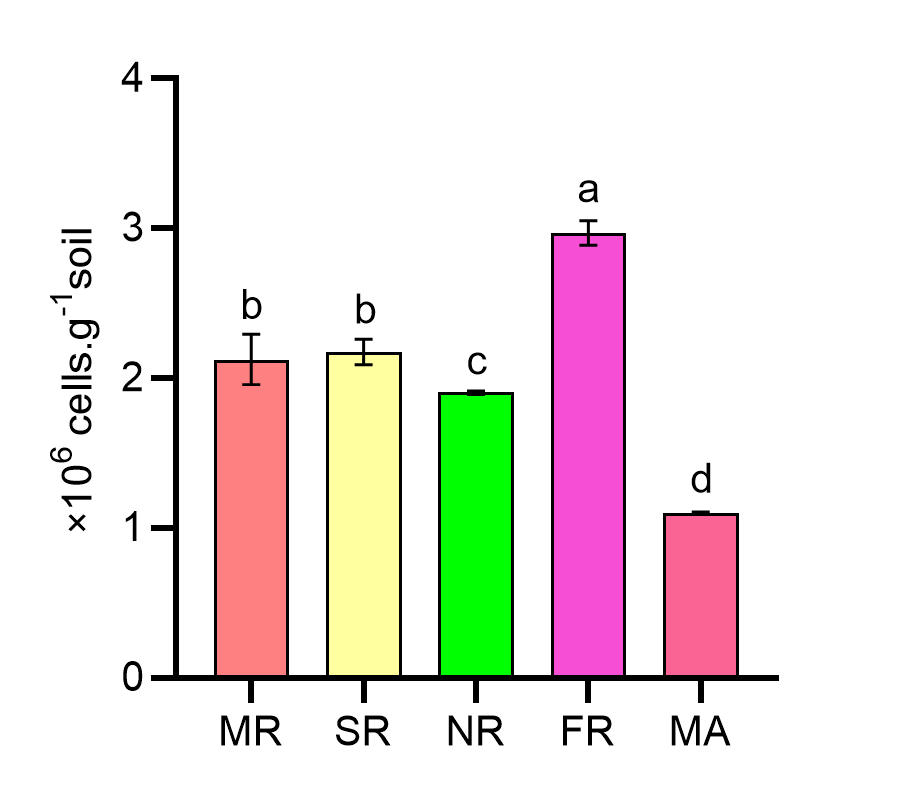


Figure S4 *Fusarium* content in rhizosphere soil of *R. glutinosa* under different treatments
